# Vehicle Trajectory Prediction via Urban Network Modeling

**DOI:** 10.3390/s23104893

**Published:** 2023-05-19

**Authors:** Xinyan Qin, Zhiheng Li, Kai Zhang, Feng Mao, Xin Jin

**Affiliations:** 1 Tsinghua Shenzhen International Graduate School, Tsinghua University, Shenzhen 518055, China; qxy20@mails.tsinghua.edu.cn (X.Q.);; 2 Research Institute of Tsinghua, Pearl River Delta, Guangzhou 510530, China

**Keywords:** trajectory big data, urban computation, trajectory prediction

## Abstract

Taxis are an important component of the transportation system, and empty taxis represent a significant waste of transportation resources. To alleviate the imbalance between supply and demand and relieve traffic congestion, real-time prediction of taxi trajectories is necessary. Most existing trajectory prediction studies focus on extracting time-series information but do not capture spatial information sufficiently. In this paper, we focus on the construction of an urban network and propose an urban topology-encoding spatiotemporal attention network (UTA) to address destination prediction problems. Firstly, this model discretizes the production and attraction units of transportation, combining them with key nodes in the road network to form an urban topological network. Secondly, GPS records are matched to the urban topological map to construct a topological trajectory, which significantly improves trajectory consistency and endpoint certainty, helping to model destination prediction problems. Thirdly, semantic information concerning surrounding space is attached to effectively mine the spatial dependencies of trajectories. Finally, after the topological encoding of city space and trajectories, this algorithm proposes a topological graph neural network to model the attention calculation with the trajectory context, comprehensively considering the spatiotemporal characteristics of the trajectories and improving prediction accuracy. We solve the prediction problems with the UTA model and also compare it with some other classical models, such as the HMM, RNN, LSTM, and transformer. The results suggest that all the models work well in combination with the proposed urban model (with a rough increase of 2%), while the UTA model is less affected by data sparsity.

## 1. Introduction

With the development of GPS technology and Internet technology, the positions of passengers and vehicles can be captured and expressed accurately in real time, and this has led to the emergence of various sophisticated services. Empty taxis represent a significant waste of transportation resources, affecting passengers, vehicles, and roads. Increasing the utilization rate of taxis through resource allocation can effectively alleviate the supply–demand contradiction as well as traffic congestion while saving the operating costs of taxis. Vehicle destination prediction is a location-based intelligent service. Given an unfinished taxi trajectory, this service can accurately predict the vehicle’s destination based on its context information and geographic spatial information, assist in taxi resource allocation, adjust the balance between supply and demand, and relieve traffic congestion.

Taxi destination prediction is not only a time series prediction problem; it also involves the utilization of spatial information. Existing methods focus on modeling the temporal dependencies of long sequences, from statistical models and machine learning models to the latest deep learning models, and there have been many advances. On the other hand, the utilization of spatial information is limited to the trajectory endpoint maps generated by endpoint clustering [1], and the modeling of road topology is also relatively imprecise, with little attention paid to key information such as city POIs, land use types, and road network nodes along the trajectory.

Trajectory big data has become more easily accessible and more widely adopted as the mobile Internet has progressed. Spatial trajectory data can accurately specify the characteristic information of a city’s operational mechanisms and help build travel prediction and recommendation systems. Zheng [2] classified the trajectories into four classes (the mobility of people, vehicles, animals, and natural phenomena), among which data information islands exist as a result of various information-collecting equipment. Thus, recent trajectory mining studies have tended to be more subtle and more specialized, combining research targets and data formats, and thereby limiting the application range of trajectory big data.

Prediction tasks have always been regarded as an open scientific issue due to the constraints of urban road network structures. To describe spatial data, two kinds of maps are typically used: vector-based maps and raster-based maps [3]. A vector-based map aims to present spatial objects with lines, points, or polygons using Cartesian coordinates, which are widely utilized in geographical information systems (GIS). Professional standards for GIS have been established for decades, and GIS services are now easy to access from specific websites such as OpenStreetMap, Google Map, Gaode Map, and Baidu Map. A raster-based map divides a study area into small grid cells to determine the temporal and spatial characteristics within these grid cells. In a typical study of trajectory data, the original study area is always vector-based and is obtained from a professional GIS website, while trajectory data as well as points of interest (POI) data are listed as a table full of coordinates. Given vector-based maps, several map-matching algorithms have been able to map the GPS records of objects into vector roads, and model topological road graphs to solve data mining tasks. The raster-based map implies an original recognition for spatial data analysis. Among researchers engaged in spatial data analyses not limited to geographical mapping, most work is carried out via map segmentation. Rectangular segmentation is one of the most common methods by which online car-hailing travel demand prediction can be used to divide a standard map into several rectangular units of equal size and the geographical characteristics of each unit can be discussed [4,5]. To reduce the querying time in the geodatabase, Gustavo Niemeyer designed GeoHash [6], a universal geocoding algorithm that divides an area into rectangular grid cells to identify the accuracy location. There are also numerous applications for other methods, such as diamond, circular, or even irregular map segmentation. Another study [7] explored human stay time patterns obtained from mobile phone data; the mobile phone signals were uniformly collected by the regularly arranged local base stations, one of which occupied its specific radiation range, leading to the division of the map into diamond-shaped cells. Polygon map segmentation methods are commonly seen in urban function analyses where the crisscross network splits a map into irregular geographical areas with different geographical functions. When studying the hidden rule of geographical points of interest, Voronoi [8] established a method to delimit the coverage of each point.

Recently, since the introduction of deep learning algorithms, there have been significant development in trajectory data mining and urban computation. Most works tend to abstract topological graphs from road maps. The application of spatial modeling to a convolutional neural network has been proposed [9,10,11]. A set of road nodes is used, and the edge set is an adjacency matrix, and vice versa. The element of the adjacency matrix is 1 if there is a link between two nodes, and 0 if there is not. A spatiotemporal feature matrix is then established and used to predict the traffic flow in the next moment. Most such efforts in traffic prediction have been used to improve the precision of deep learning models such as multilayer perceptron [12], recurrent neural networks (RNNs) [13], long short-term memory (LSTM) [14], gated recurrent units (GRUs) [15], and so on. To excavate the spatial correlations of topological maps, researchers have been trying to combine RNNs with convolutional neural networks (CNNs) [16], and this has enabled them to efficiently capture hidden spatial dependencies. Lai [16] proposed a long-and-short-term temporal network (LSTN), replacing the RNN part with a stronger LSTM. With the development of the transformer [17] in computer vision (CV) [18,19] and natural language processing (NLP) [20,21,22], issues with sequence prediction were soon resolved using these attention-based models. To leverage transformers in spatial data analysis, great efforts have been made to design a GNN-based [23,24,25,26] attention mechanism in the graph domain.

As with traffic flow prediction (a macro perspective), efforts have been made to study the behavior patterns of pedestrians (a micro perspective). The rapid popularization of mobile technology such as GPS and Wi-Fi has made it possible to obtain detailed and continuous geo-location data, and this has led to the development of location-based social networks (LBSNs) [27]. Unlike raster maps or vector maps, LBSNs view maps as a set of semantic points which only contain the most valuable information concerning location and types of land use. LBSNs record users’ check-in and check-out times on specific semantic points and combine these with their current emotions, the weather conditions, and various other relevant information. With the modeling of LBSNs, user behavior can be described as a spatiotemporal sequence listed by semantic points and forming a user behavior matrix. Thus, user behavior patterns can be studied with various prediction models and recommendation systems.

Looking at the above commonly used map modeling methods, we can see that map modeling has reached a near-point level, and research on sequence prediction models has evolved into multiple variations of attention mechanisms and graph modeling. However, research on destination prediction tasks is still limited to the extraction of time series features, and the capture of spatial information is far from sufficient. Therefore, this section proposes an updated mapping and trajectory modeling method based on urban topology modules, sequence self-attention mechanisms, and graph neural network modeling to fully learn the spatiotemporal characteristics of trajectory data.

This paper proposes a novel modeling method for urban spaces to support the topological trajectory definition of vehicles which can be useful in the prediction of trajectory and help in the construction of destination recommendations. The entrances of neighborhoods and large buildings are extracted as topological points and subsequently concatenated into a graph with the assistance of intersection points. In this way, vehicle trajectory can be determined via the sorting of finite topological points, and this helps define the prediction task.

In summary, this method includes the following innovative features:

- The production and attraction units of transportation are discretized to form an urban topological network. The consistency and determination are significantly improved to represent a trajectory.
- The urban topological encoding part calculates the semantic vector of points through urban modeling and adds it to the trajectory as an extension dimension, effectively mining the spatial dependencies of trajectories.
- The spatiotemporal attention part comprehensively considers the spatiotemporal characteristics of trajectories and improves prediction accuracy.

This paper is organized as follows: Section 2 describes urban network modeling, including the establishment of urban topology maps, the generation of semantic vectors, and the definition of topological trajectories. This section also introduces the relevant research areas and the specific processing steps for maps and GPS data. Section 3 defines our trajectory prediction task, introduces the UTA model in detail, and describes the main deep learning model utilized in this prediction task. Section 4 outlines the evaluation metrics and designs of our experiment. The performances of the various models are also presented in this section. Finally, in Section 5, we present our conclusions.

## 2. Urban Modeling and Trajectory Definition

### 2.1. Study Area

**Figure 1:**
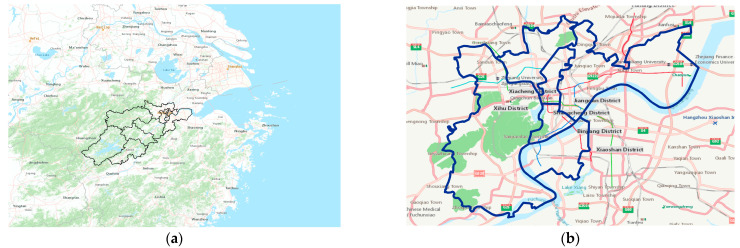
The study area. (**a**) The position of Hangzhou in China. (**b**) The core business zone of modern Hangzhou.

Our GPS records data pertain to the core urban area of Hangzhou City, Zhejiang Province, China, and the position is shown in Figure 1. Consequently, Hangzhou City is taken as an example to verify our urban modeling. Hangzhou is the capital of Zhejiang Province and is located at 118.33–120.50 E longitude, and 29.18–30.51 N latitude. Hangzhou has a long-term resident population of nearly 1.2 million and a gross domestic product (GDP) of 1610 billion yuan; it thus serves as one of the most important cities in the Yangtze River Delta. Compared with the complex mountainous areas in the west, eastern Hangzhou has a higher population and more urban land. In this article, the following six core districts are used as the study area: Shangcheng District, Xiacheng District, Jianggan District, Gongshu District, Xihu District, and Binjiang District. These six districts comprise the old town of Hangzhou, which is still the core business zone of modern Hangzhou, and they are crowded with efficient roads, rail links, tunnels, and flyovers.

### 2.2. Generation of Semantic Points

A map can be produced simply with nodes and links. In this section, further semantic details will be added using the Gaode open website (one of the best digital map providers in China) to optimize the map.

Urban networks, in which districts and neighborhoods use vehicles to provide various essentials, are the lifeline of a city. These semantic areas are always called areas of interest (AOI) or are focused on a point called a point of interest (POI). One AOI always contains many POIs. In this paper, 2785 AOIs were collected using Gaode data from parking lots leading to main roads. According to the parking lot entrance/exit gates, all AOIs join main roads via inject points. When the topological lines (described in step 4 of Section 2.2) are interrupted at these inject points, a new semantic map is generated.

Where the land use and location were too minor to depict an AOI, we tried to count the number of different POIs in the area as an added attribute. Gaode divides all the POIs into the following 20 categories: automobile service POIs, automobile sale POIs, automobile maintenance POIs, motorcycle service POIs, catering POIs, shopping POIs, life service POIs, recreational and entertainment POIs, healthcare POIs, accommodation POIs, tourist destination POIs, residential POIs, government POIs, educational and cultural POIs, transportation POIs, finance and insurance POIs, cooperative and business POIs, road ancillary facility POIs, communal facility POIs, and public event POIs. To make them consistent with the semantic points, the intersections obtained in Section 2.2 were added to these 20 categories. POIs within 1000 m of the center of the intersection were counted. The fields of the points are explained in Table 1.

### 2.3. Generation of Topo Map 

This section described how the original maps were processed to obtain the connections of the intersections. We used the same method that most researchers use, i.e., we obtained map data from OpenStreetMap and downloaded network data in the form of spatial line features. Table 2 below lists the line feature attributes.

To deal with the mixed and disorderly network, four steps were used to simplify the original data.

Step 1: only trunks, tracks, and paths are valuable for a vehicle, so 14 classes were picked out of the 27 primary “fclass” classifications.

Step 2: Maps from OpenStreetMap are used to draw many parallel lines within individual roads, especially major intersections, to describe every passable path. In this paper, however, the topology links of the network are focused on since only one line is needed to describe each road. Consequently, buffers with a width of 20 m are generated on both sides of every road line to cover the inner lines of the roads, transforming the spatial line feature into a spatial polygon feature. After vectorization editing, approximate road center lines are extracted from the road polygon.

Step 3: It is observed that the networks obtained in step 2 contain certain long paths, both straight and curved, and this is unsuitable for fitting the trajectory. In this step, paths longer than 2000 m are highlighted and then divided into sub-paths not exceeding 1000 m, as this is beneficial for traffic observation.

Step 4: The above steps successfully extract every independent road and endpoint, where every endpoint proves to be the center of one intersection. The semantic points in Section 2.2 are identified and joined to the nearest roads (determined in Step 3). These roads and endpoints together constitute the vertices of a new road graph and interrupt the roads from step 3, forming a new adjacency matrix.

In this way, a graph G=(V,E) was established, where V represents the set of all vertices and E represents an adjacency matrix according to the roads. The Figure shows the procedure of each step after extraction. The original 26,659 lines and 174,947 points were then reduced to only 4211 intersections and 2758 projection points, connected with 9055 edges.

**Figure 2:**
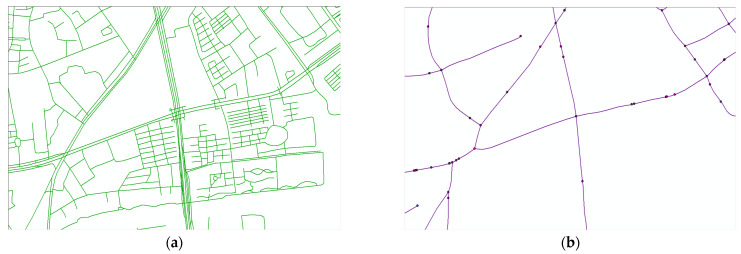
The processes of developing a topological map. (**a**) The original map of an intersection. (**b**) The final topological map.

The final topological map of Hangzhou is shown in Figure 2. Figure 2a shows the original map obtained from OSM and is full of redundant lines, e.g., lanes, paths, links, and steps. Figure 2b shows the final topological map, processed through the above steps. In the topological map, the buildings and communities serving as urban functional units are abstracted into a point and projected onto the road network. These projected points, together with the road hubs, form the black points in the figure. The topology edges are realized by simplifying the original map’s road center lines, which are shown as purple lines in the figure.

### 2.4. Trajectory Processing

Our dataset was obtained from Hangzhou Transportation Satellite Position Application Co., Ltd, Hangzhou, China. The dataset consists of 8,507,317 global position system (GPS) records, spanning two weeks from 26 October to 4 November 2021 and covering 31,235 electric and hybrid vehicles. Each record contains three types of information: spatial information, temporal information, and vehicle information. The time and location of sampling are typical spatiotemporal data. The vehicle information includes the vehicle identification number (VIN), battery model, platform name, vehicle state, message type, motor type, and various other pieces of information used for monitoring vehicle states. The fields are listed in Table 3.

The original trajectory data seem essentially to be a series of non-uniform sampling points with rich semantic information hidden and stored in JSON format. Considerable irrelevant information is added along with the spatiotemporal coordinates, and this significantly increases the difficulty and cost of storage. Trajectory data mining only requires spatiotemporal information to conduct various prediction and controlling tasks based on a topological graph. In this context, a large amount of irrelevant information leads to low indexing efficiency. Additionally, the coordinate encryption design as well as the inherent error makes it necessary to match the GPS data to a real map for further analysis.

Section 2.2 and Section 2.3 help construct a novel topo map full of semantic points. The unique map-matching method aims to redescribe the trajectory with a sequence. The simple nearest neighbor algorithm is used to match each GPS point with a semantic point or intersection within a range of 1000 km. The point lists are then sorted into chronological order and duplicate points are eliminated to identify the stay state of a vehicle. Finally, a new trajectory sequence is obtained.

## 3. Trajectory Prediction

### 3.1. Problem Definition

Following the topo map generating process, we finally obtain a topological map which describes a city, written as G=(P,E), where point set $P=\{p_{1},p_{2},\ldots ,p_{N}\}$ represents N points selected in Section 2 and E represents the set of topological relationships. Each point has 22 attributes, including longitude, latitude, name, and number of POI categories within 1 km. In this way, the trajectory of the vehicle $v_{i}$ can be described as a vector $traj_{v_{i}}$:

(1) $${traj}_{v_{i}}=[(p_{1},t_{1}),(p_{2},t_{2}),\cdots ,(p_{n},t_{n})]$$

In the formula, p represents one point in P, t represents the time when the vehicle arrives at this point, and a tuple $\left(p_{n},t_{n}\right)$ represents the arrival of the vehicle $v_{i}$ at point $p_{n}$ and at time $t_{n}$.

This trajectory is only a projection calculated using GPS records representing the spatiotemporal location information of the taxi; it does not include the running and stopping states of the vehicle. Therefore, we define the trip ${traj}_{v_{i}}^{j}$, which is essentially the $j^{th}$ sub-trajectory of vehicle $v_{i}$, as follows:

(2) $${traj}_{v_{i}}^{j}=[(p_{o},t_{o},st_{o}),\cdots ,(p_{d},t_{d},st_{d})]$$

The formula uses (p,t,st) to describe the vehicle trip, and the subscripts represent the starting point, intermediate point, and ending point. Unlike the determination of the topological trajectory, the determination of the topological sub-trajectory adds the st item, which represents the weighted stay time of the vehicle at that point. For the stopping time $st_{j}^{i}$ of vehicle $v_{i}$ at point $p_{j}$, the formula is as follows:

(3) $${st}_{j}^{i}=\frac{t_{j+1}^{i}-t_{j}^{i}}{d(p_{j-1},p_{j+1})}$$

The function d(·,·) in the formula calculates the distance between two points on the urban topological map. The physical meaning of the formula is that the vehicle’s stay time is proportional to the time spent by the vehicle at that point and inversely proportional to the coverage range of that point.

In practice, a threshold value of 15 min/1 km is given. Trajectory states higher than this value are assumed to indicate that the vehicle is staying at the functional point. The trajectory is segmented into several trips from the starting point to the ending point.

The destination prediction problem is essentially to give N points and time records that a vehicle has passed through from the start to the current position, and the expected model predicts the endpoint of the vehicle for this segment of the trip. In the experiment, the trajectory sequences are input as time data, and the map data are input as spatial data. During the superimposition and gradient propagation processes, the model scores and ranks all the points. The highest-ranked functional point can be considered the vehicle’s predicted destination according to the model. Assuming the input matrix is X, representing the total vehicle trip matrix, and the total number of vehicles is M, the output vector Y represents the next functional point that the model predicts these vehicles will pass through. The formula is as follows:

(4) $$X=\left(\begin{array}{c} \left(p_{v_{1}}^{1},t_{1}\right),\cdots ,\left(p_{v_{1}}^{K},t_{K}\right)\\ \ldots \\ \left(p_{v_{M}}^{1},t_{1}\right),\cdots ,\left(p_{v_{M}}^{K},t_{K}\right) \end{array}\right)$$

(5) $$Y=\left[p^{v_{1}},\ldots ,p^{v_{M}}\right]$$

**Figure 3:**
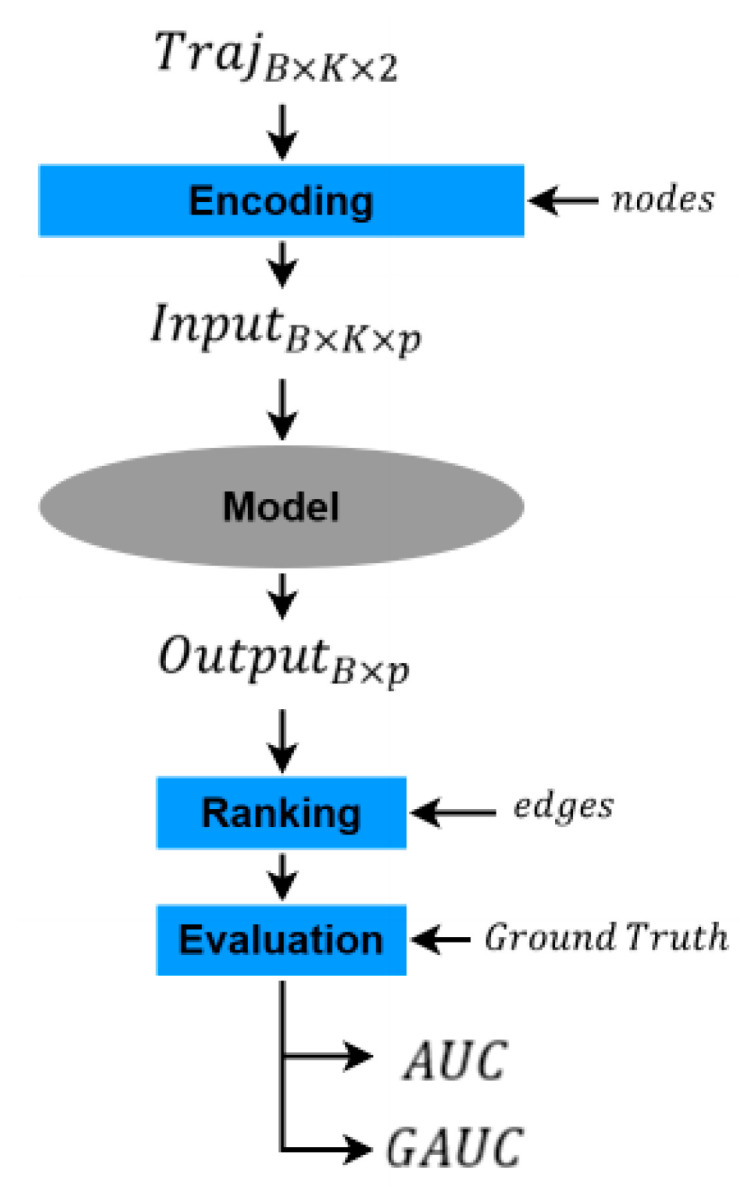
Vehicle trajectory prediction process.

After urban topological modeling and trajectory modeling, the interaction between vehicles and locations is similar in form to the interaction between users and items in recommendation systems. Figure 3 depicts the main process of vehicle trajectory prediction. The trajectory matrix has three dimensions. B represents the batch size, K represents the length of the trajectory, and the final two represent the point number and the stay time at the point. After the encoding layer, the data are transformed into a three-dimensional feature matrix with dimensions of B, K, and p. p=20 is the length of the semantic vector, and the specific value is the semantic component multiplied by the weight. The weight is the normalization of the time spent at the functional point and the ratio of the functional radius. The model was evaluated using the area under curve (AUC) and group AUC (GAUC) of the receiver operating characteristic curve of the participant.

### 3.2. Overview

To more effectively extract spatiotemporal dependencies from vehicle trajectories, we propose an urban topology-encoding spatiotemporal attention network (UTA) to predict taxi endpoints in real time. The UTA primarily consists of an urban topology module and an attention calculation module. The urban topology module highlights the semantic and traffic dependencies of vehicle journeys by re-modeling maps and trajectories, learns traffic topology information through point encoding, edge encoding, and space encoding, and learns surrounding semantic information through semantic vector calculation. The attention calculation module decomposes the trajectory matrix into query vectors, key vectors, and value vectors, determines important key feature points through self-attention calculation, and learns the time information of trajectories.

**Figure 4:**
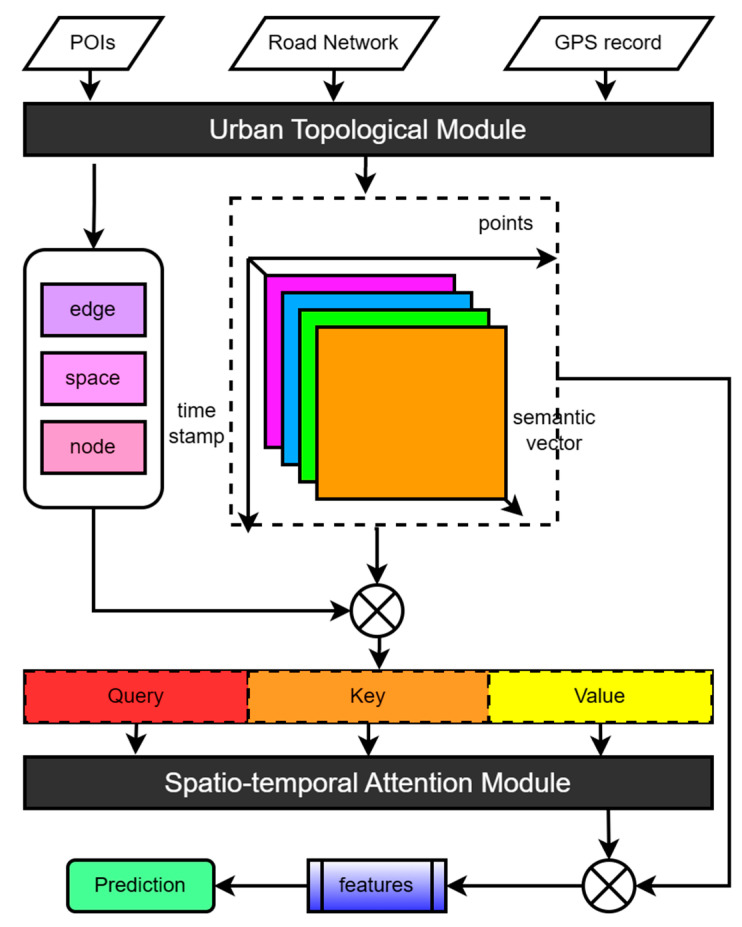
Architecture of the proposed UTA.

The basic architecture of the urban topology-encoding spatiotemporal attention network is presented in Figure 4. It mainly consists of two parts: the urban topology module and the spatiotemporal attention calculation module. These two modules will be introduced and explained below.

### 3.3. Urban Topology Module

The urban topology module performs the input function of the model. This includes the generation of the urban topology graph, the matching of GPS records to topology trajectories, the generation and normalization of sub-topology trajectories, and the semantic vector encoding of feature points. The process of generating the urban topology graph has been discussed in the previous section, and the matching of the topology trajectory and the generation of sub-topology trajectories have also been introduced. This section focuses on the introduction of the semantic vector encoding operation.

It is necessary to quantify the interactive relationships between vehicles and destinations, which look the same as the interactions between users and items. One-hot coding [28] involves categorizing information into binary columns (either 0 or 1). This provides many dimensions for deep learning models, resulting in a waste of computing resources. Abundant methods have been proposed to represent a classification column, and these include target coding [29], leave-one-out coding [30], Bayesian target encoding [31], and many others. In addition to feature densification, the points in this topo map contain rich semantic information, and the number of POIs has been counted in Section 2.2. Since there may be dozens of restaurants but only one school or hospital in an area, it seems necessary to define a semantic vector to quantify the semantic feature within one topo point. For point i and POI j, the semantic vector can be obtained as follows:

(6) $$f_{i}=\left[w_{1},\ldots ,w_{j},\ldots ,w_{20}\right]$$

where i varies from 1 to M and $w_{j}$ indicates the weight of the j th POI, ranging from 1 to 20. $w_{j}$ can be calculated as follows:

(7) $$w_{j}=\frac{n_{j}}{\sum n_{k}}\times log\frac{\left|P\right|}{\left|\{p|p\in PandphasPOIj\}\right|}$$

where $n_{j}$ means the number of POI j in all points. The operation |·| calculates the number in the set. p and P have been discussed in Section 3.1.

### 3.4. Spatiotemporal Attention Module

In time series, the correlation between samples is used to calculate the attention of samples. In graph networks, the correlation between points is specified by edges and the importance of points is measured by in-degree and out-degree. This centrality of points is a very important parameter for understanding the graph. Introducing point centrality can effectively enable the urban topology-encoding spatiotemporal attention network to learn the graph structure.

The urban topology-encoding spatiotemporal attention network introduces the calculation of point centrality as an additional signal added to the network. The features of each point are encoded by inputting them into a point-encoding module, as is shown in the following formula:

(8) $$h_{i}^{0}=x_{i}+z^{-}+z^{+}$$

where $z^{-}$ means the in-degree vector and $z^{+}$ means the out-degree vector. These trainable vectors are all able to describe the correlation and importance of the semantic points.

One significant advantage of the self-attention mechanism is its global receptive field, by which each token in each layer of the neural network can learn information from all positions and then calculate feature vectors. However, this feature is based on accurate position encoding for all positions. For sequence data, simple trigonometric functions can achieve unique positioning encoding. However, for graphs, the arrangement of points differs significantly from that of sequence data; the points are instead located in a multidimensional space connected by edges. In this section, the spatial encoding method is adopted to obtain a mapping $\varphi \left(v_{i},v_{j}\right):V\times V\to R$ to describe the relationship between point i and point j. The φ function is defined as the shortest path distance (SPD) between two points, and if the two points are not adjacent, it is set to −1. In this case, the softmax scores in the self-attention mechanism can be described as follows:

(9) $$Att=softmax\left(\frac{(h_{i}W_{Q}){\left(h_{i}W_{K}\right)}^{T}}{\sqrt{d}}+b_{\varphi \left(v_{i},v_{j}\right)}\right)V$$

where $W_{K}$, $W_{Q}$, and b are all learnable. $h_{i}$ has been discussed above.

With this setting, each point in the self-attention mechanism can participate in the calculation of other points in the graph, expanding the receptive field to the entire graph. This participation is adaptive, and the degree of involvement is determined by the distance relationship between two points, which is realized by the gradient of the weights. The bias setting also ensures that points closer to the computing point will have a greater weight.

In a graph, points are connected by edges, which also express quantifiable structural information necessary for the model, such as type, length, and direction. Edge encoding aims to model these edge properties, and there are two commonly used methods to achieve edge encoding: one is to add edge information to the endpoint of the edge [32,33], and the other is to cross edge information with point information during the aggregation phase. Generally speaking, the essence of these methods is to describe edges using points, avoiding the most naive features of the edges.

The attention mechanism requires correlation calculations between every pair of points, and these include not only the correlation between points but also the involvement of the edges connecting these two points. In spatial encoding, the SPD is used to describe the correlation between points, and the edges that make up the shortest path also require a representation layer. The participation of the edge in numerical terms is represented by an additional bias term. $A_{ij}$ represents the value inside the softmax of the attention mechanism, and after performing edge encoding, the calculation formula is transformed into:

(10) $$A_{ij}=\frac{(h_{i}W_{Q}){\left(h_{i}W_{K}\right)}^{T}}{\sqrt{d}}+b_{\varphi \left(v_{i},v_{j}\right)}+\frac{1}{N}\sum_{n=1}^{N}x_{e_{n}}{\left(w_{n}^{E}\right)}^{T}$$

where $x_{e_{n}}$ is the feature vector of the *n*th edge, $SPD_{ij}=(e_{1},e_{2},\cdots ,e_{N})$ is the shortest path between point i and point j, $w_{n}^{E}$ is a trainable weight vector used to adjust the residual term, and $d_{E}$ is the dimension of the weight vector.

## 4. Prediction Results

This section presents the vehicle trajectory prediction of the UTA and other baselines. The results are evaluated using AUC and other metrics. We use GPS records from the Hangzhou center district to train and test. The first thirty records are used to predict the next destination.

### 4.1. Evaluation Metrics

For these sorting tasks, area under the curve (AUC) [34] is one of the most suitable methods for evaluating the performance of the models. In this paper, AUC and group AUC (GAUC) are both taken into account. AUC indicates the area value under the receiver operating characteristic curve, and it represents the probability that the predicted score of the positive destination is higher than that of the negative destination. A classification model can calculate the probability that a sample belongs to each category (destination or not), and the point with the highest score will win the sorting and be used as the prediction. The physical significance of AUC caters to the principles of the classification model and reflects the efficiency of the models at a deep level. Since the distance and time required by each vehicle varies with a range of 10,000, GAUC is also used to evaluate the model at a subtle level. GAUC tests the model’s ability to achieve a personalized destination prediction for each vehicle. The formulas are as follows:

(11) $$AUC=\frac{\sum_{M\times N}I(p_{positive},p_{negative})}{M\times N}$$

(12) $$I\left(p_{positive},p_{negative}\right)=\left\{\begin{array}{c} 1,p_{positive}>p_{negative}\\ 0.5,p_{positive}=p_{negative}\\ 0,p_{positive}<p_{negative} \end{array}\right.$$

(13) $$GAUC=\frac{\sum_{v_{i}}w_{v_{i}}\times AUC_{v_{i}}}{\sum w_{v_{i}}}$$

where M and N are the pair of randomly selected samples and $w_{v_{i}}$ is the number of points the vehicle $v_{i}$ has passed by.

In addition, accuracy, root mean squared error (RMSE), and mean absolute error (MAE) are used as evaluation criteria to quantify the differences between the predicted distances and the actual distances. The accuracy is determined by the proportion of correctly sorted targets. The others are defined as follows:

(14) $$RMSE=\frac{1}{n}\sum_{i=1}^{n}\left|d(y_{i},{\hat{y}}_{i})\right|$$

(15) $$MAE=\sqrt{\frac{1}{n}\sum_{i=1}^{n}{\left(d\left(y_{i},{\hat{y}}_{i}\right)\right)}^{2}}$$

where the function $d(y_{i},{\hat{y}}_{i}$) in the formulas calculates the distance between the real destination $y_{i}$ and the predicted destination ${\hat{y}}_{i}$, and n is the number of trajectories.

### 4.2. Baselines

We compared the UTA discussed in Section 3 with various deep learning models, including the classic HMM model, an attention-based model, a GNN-based model, and many other state-of-the-art models.

- Hidden Markov model (HMM) [35]: a classic probabilistic model for time series correlation that is used to calculate observation probabilities by assuming that a vehicle’s current trip depends only on the previous trip.
- Recurrent neural network (RNN) [13]: a specially designed neural network for dealing with sequence input with recurrent layers to learn the former input.
- Long short-term memory (LSTM) [14]: a novel model based on the original RNN designed to capture more information over a longer period.
- Graph convolutional network (GCN) [36]: A model that makes use of the adjacency matrix to generate a feature matrix and is then imputed into the convolution layer. It has shown great potential in the excavation of graphs.
- Transformer [17]: a typical attention-based model that has been proven more efficient in sequence prediction than the RNN structure.

### 4.3. Experimental Results

As was described in the previous section, the POI semantic vector length was set to 20 in the experiment. An Adam optimizer with a learning rate of 0.0001 was used. During each iteration, 40% of the neurons were randomly frozen to achieve masking. Batch sizes of 20 and 200 were used in the experiment. The next destination was predicted based on the first 30 trajectory points, and the empty point was padded with a 0.

The HMM model and the UTA’s topology point generation, map matching, geographical semantic attachment, and other modules were completed locally, while the other neural network models used GPU acceleration. Hyper-parameters were set following the default settings of the baselines. It took 73.49 minutes to train the RNN and 103.90 minutes to train the LSTM. This significant time consumption is one of the limitations of such neural networks. It took 29.31 minutes to train the GCN, implying that extracting spatial dependencies requires little time. The transformer took 35.66 minutes to train, and the UTA took 54.34 minutes. The results show that the attention mechanism is superior in both accuracy and time consumption compared with traditional recurrent neural networks. However, the UTA model takes more time to learn spatiotemporal dependencies, resulting in higher time consumption than the pure self-attention mechanisms.

Table 4 compares the performance of each model in indicators such as accuracy, RMSE, and MAE. Urban topology modeling abstracts urban space into a set of finite points, and the vehicle destinations are also constrained within these points. However, as destinations, these topological points contain inherent positional coordinate information. Therefore, the experiment compared the positional errors of the destination prediction results to determine the performance of each model. The results show that all of the models performed well in terms of precision. However, regarding positional errors, the performance of the time series prediction models was generally inferior to that of the spatial models. It is difficult for time models to extract spatial dependencies from the data, and the distance factor of the destination is magnified during the prediction process. Therefore, the discrepancies in distance of the failed samples is more obvious. As a result, the RNN, the LSTM, the transformer, and the other models performed worse than spatial models in terms of RMSE and MAE. Due to the effective semantic attachment of topology modeling to trajectories, the HMM’s performance was greatly improved, surpassing that of the RNN and LSTM in terms of RMSE and MAE.

Urban topology modeling limits trajectory behavior to fixed topological points, significantly reducing uncertainty in the trajectory and greatly improving absolute error, relative error, accuracy, and other indicators. On the other hand, the trajectory itself belongs to discrete data, and the destination is a significantly imbalanced sample, making accuracy evaluation more difficult. Therefore, more commonly used sorting-based evaluation metrics, such as AUC and GAUC, were used to further evaluate the models.

Table 5 presents the trajectory prediction performance of the urban topology-encoding spatiotemporal attention network and the baseline model. The urban topology-encoding spatiotemporal attention network achieved the best performance with an AUC of 0.9597, which is 2.37% better than that of the transformer. The GCN model performed similarly to the transformer in terms of AUC, indicating that although the GCN learns more about spatial structures, the powerful time-dependency mining ability of the transformer enables it to perform better. In terms of GAUC, the urban topology-encoding spatiotemporal attention network achieved a value of 0.9554, ranking first among all the models. This value is 1.71% higher than that of the transformer and 2.37% higher than that pf the GCN. Overall, the performance of all the deep learning models was better than that of the traditional regression model, the HMM. These results indicate that attention-based models completely outperform RNN-based models in destination prediction tasks. The GCN models that captured spatial correlation performed better than classical sequence prediction models, with a roughly 0.02 increase in performance, but the improvement in GAUC performance was not significant, indicating that the GCN’s ability to capture sequence information is inferior. The sequence prediction models (such as the RNN and the transformer) produced stable performances for both short- and long-term predictions, and the attention-based models generally outperformed the RNN-based models in prediction tasks.

Table 6 presents the results of the ablation experiments performed on the urban topology-encoding spatiotemporal attention network. “TA” represents time attention, “SA” represents spatial attention, and “UTE” represents urban topology encoding. “w/o” indicates the experimental data obtained when the module was removed, while “Ours” represents the urban topology-encoding spatiotemporal attention network proposed in the paper. 

The ablation experiment results reveal the following facts:

- Urban topology encoding has the greatest impact on GAUC. The SA and UTE modules work together for spatial dependency learning, with SA focusing on spatial information calculation and UTE focusing on trajectory definition and semantic information recognition. Removing the UTE module results in a 0.04 decrease in GAUC, indicating that the UTE module highlights the spatial dependence of trajectories and helps to personalize destination prediction for different vehicles and trips.
- Attention mechanisms have significant advantages in learning temporal dependencies. The experimental results show that the model’s AUC results significantly decline when the time attention calculation module is removed, with a decrease of nearly 0.05 compared with the urban topology-encoding spatiotemporal attention network, resulting in a performance similar to that of the RNN model. The attention mechanism autonomously selects key information from long sequence data, effectively handling sparse trajectory data, and this makes it the preferred model for time-series prediction tasks.

**Figure 5:**
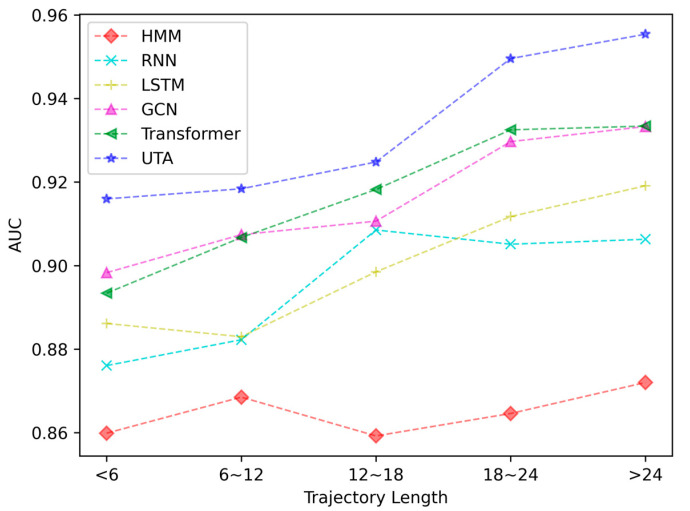
Comparisons of AUC performance under different trajectory lengths.

It should be noted that the shortest topology sub-trajectory length for new energy vehicles in Hangzhou is 1, and the maximum length is 33. The trajectory length directly affects the performance of a trajectory prediction model. Shorter trajectory data contain less semantic information, and this is more likely to cause overfitting of the model. Therefore, we divided the trajectories into five groups according to their length with an interval of six, and we conducted prediction experiments on each group of trajectories. The parts of the trajectories with a length greater than 24 constitute the entire experiment, with a trajectory length of 30. The experimental results are shown in Figure 5.

It can be seen from the figure that as the trajectory length increases, the prediction accuracy of each model also increases, with UTA performing the best at each length. The performances of the transformer and the GCN are the closest to that of the UTA. When the trajectory is short, the transformer can only learn limited sequence information, while the GCN can learn sufficient information from the urban topology road network. When the trajectory is long, the spatial performance of GCN is weakened, and the transformer demonstrates strong sequence induction ability. In summary, our UTA model shows significant performance improvement compared with other models for both long and short trajectory sequences.

## 5. Conclusions

To improve the accuracy of taxi destination predictions and enable better traffic resource scheduling to alleviate traffic congestion, this paper proposes an urban topology-encoding spatiotemporal attention network. Based on the urban topology system and urban topology maps, this paper establishes topological trajectories and sub-trajectories, reshapes the destination prediction task, and presents the results of comprehensive comparative experiments and ablation experiments.

The experimental results show that the urban topology-encoding spatiotemporal attention network performs better than classic models such as the HMM, RNN, LSTM, GCN, and transformer in both long-term and short-term predictions, demonstrating the potential of attention mechanisms and urban topology maps. The results of the ablation experiments show that the attention calculation network contributes significantly to the overall model accuracy, increasing the AUC from 0.9037 to 0.9554. Urban topology modeling greatly reduces the need for storage and improves semantic density in the abstraction expression of trajectories and maps, and the improvement in GAUC is particularly evident. Graph modeling and spatial attention calculation mechanisms are important supplements to the model. Looking to the future, we believe that advanced trajectory prediction models can help establish personalized recommendation systems and path planning systems.

## Figures and Tables

**Table 1:** Vertice fields and description.

Field	Description
Location	The longitude and latitude of the center of the point.
Semantic vector	A vector with 20 elements among the ranging area.
Type	Intersection or inject point marking the origin.
FID	The only recognition code for the point.

**Table 2:** Types of roads obtained from OSM.

fclass	Description
Tertiary	Urban branch roads
Tertiary link	Ramp roads
Residential	Roadways in residential areas
Unclassified	Waterside areas or airports
Secondary	Secondary urban roads
Secondary link	Secondary ramps or interchanges
Primary	Primary urban roads
Primary link	Primary ramp or interchanges
Motorway	Urban freeways
Motorway link	Motorway ramps or interchanges
Trunk	Elevated expressways, airport arrival expressways, or bridge expressways
Trunk link	Ramps or trunk interchanges
Track	Paths in suburbs, villages, industrial and mining areas, fields, or forests
Track grade1	Paths in suburbs, villages, industrial and mining areas, fields, or forests
Track grade2	Paths in suburbs, villages, industrial and mining areas, fields, or forests
Track grade3	Paths in suburbs, villages, industrial and mining areas, fields, or forests
Track grade4	Paths in suburbs, villages, industrial and mining areas, fields, or forests
Track grade5	Paths in suburbs, villages, industrial and mining areas, fields, or forests
Bridleway	Exclusive roads inside stadiums
Living street	Roadways in residential areas and parks
Path	Roadways in residential areas and parks
Service	Roadways in residential areas, parks, railway stations, parking lots, public transport hubs, or public building entrance areas
Footway	Walkways by the waterside, parks, squares, universites, stations, or sidestreets
Pedestrian	Pedestrian streets in parks, squares, or residential areas
Steps	Steps on footbridges, squares, public buildings, or mountains
Cycleway	Non-motorized lanes in by the waterside or in park areas
Unknown	Roadways by the waterside, campus squares, or country roads

**Table 3:** Trajectory data fields and descriptions.

Field	Description
Vehicle state	The code for identifying start, flameout, error, etc.
Charging state	The code for identifying parking, driving, battery charge.
Operating mode	The code for identifying electric, petrol, or hybrid vehicles.
VIN	The unique recognition code of a vehicle.
Timestamp	The time at which GPS is recorded.
Location	The longitude and latitude of the GPS record.

**Table 4:** The efficiency of the models.

Model	HMM	RNN	LSTM	GCN	Transformer	UTA
Accuracy	0.953	0.960	0.974	0.975	0.991	0.994
RMSE/m	1456.40	2063.98	1855.15	983.02	1706.31	600.28
MAE/m	572.91	1099.39	931.61	286.01	543.72	179.46

**Table 5:** The AUC and GAUC of the models.

Model	HMM	RNN	LSTM	GCN	Transformer	UTA	Improv.
AUC	0.8720	0.9063	0.9191	0.9333	0.9334	0.9554	2.37%
GAUC	0.8923	0.9416	0.9237	0.9334	0.9436	0.9597	1.71%

**Table 6:** The performances of the various coding methods.

Model	w/o TA	w/o SA	w/o UTE	Ours
AUC	0.8720	0.9063	0.9191	0.9333
GAUC	0.8923	0.9416	0.9237	0.9334

## Data Availability

The GPS datasets analyzed in this study are available from the corresponding author on reasonable request. The GIS Hangzhou map data used in this article were obtained from https://www.openstreetmap.org (accessed on 19 July 2022). The Hangzhou POI data were obtained from https://lbs.amap.com/ (accessed on 13 August 2022) and are available from the corresponding author on reasonable request.
